# Inhibition of Janus kinase signaling during controlled mechanical ventilation prevents ventilation-induced diaphragm dysfunction

**DOI:** 10.1096/fj.13-244210

**Published:** 2014-07

**Authors:** Ira J. Smith, Guillermo L. Godinez, Baljit K. Singh, Kelly M. McCaughey, Raniel R. Alcantara, Tarikere Gururaja, Melissa S. Ho, Henry N. Nguyen, Annabelle M. Friera, Kathy A. White, John R. McLaughlin, Derek Hansen, Jason M. Romero, Kristen A. Baltgalvis, Mark D. Claypool, Wei Li, Wayne Lang, George C. Yam, Marina S. Gelman, Rongxian Ding, Stephanie L. Yung, Daniel P. Creger, Yan Chen, Rajinder Singh, Ashley J. Smuder, Michael P. Wiggs, Oh-Sung Kwon, Kurt J. Sollanek, Scott K. Powers, Esteban S. Masuda, Vanessa C. Taylor, Donald G. Payan, Taisei Kinoshita, Todd M. Kinsella

**Affiliations:** * Rigel Pharmaceuticals, South San Francisco, California, USA; and; † Department of Applied Physiology and Kinesiology, University of Florida, Gainesville, Florida, USA

**Keywords:** muscle wasting, mitochondria, oxidative stress, STAT3

## Abstract

Controlled mechanical ventilation (CMV) is associated with the development of diaphragm atrophy and contractile dysfunction, and respiratory muscle weakness is thought to contribute significantly to delayed weaning of patients. Therefore, therapeutic strategies for preventing these processes may have clinical benefit. The aim of the current study was to investigate the role of the Janus kinase (JAK)/signal transducer and activator of transcription 3 (STAT3) signaling pathway in CMV-mediated diaphragm wasting and weakness in rats. CMV-induced diaphragm atrophy and contractile dysfunction coincided with marked increases in STAT3 phosphorylation on both tyrosine 705 (Tyr705) and serine 727 (Ser727). STAT3 activation was accompanied by its translocation into mitochondria within diaphragm muscle and mitochondrial dysfunction. Inhibition of JAK signaling during CMV prevented phosphorylation of both target sites on STAT3, eliminated the accumulation of phosphorylated STAT3 within the mitochondria, and reversed the pathologic alterations in mitochondrial function, reduced oxidative stress in the diaphragm, and maintained normal diaphragm contractility. In addition, JAK inhibition during CMV blunted the activation of key proteolytic pathways in the diaphragm, as well as diaphragm atrophy. These findings implicate JAK/STAT3 signaling in the development of diaphragm muscle atrophy and dysfunction during CMV and suggest that the delayed extubation times associated with CMV can be prevented by inhibition of Janus kinase signaling.—Smith, I. J., Godinez, G. L., Singh, B. K., McCaughey, K. M., Alcantara, R. R., Gururaja, T., Ho, M. S., Nguyen, H. N., Friera, A. M., White, K. A., McLaughlin, J. R., Hansen, D., Romero, J. M., Baltgalvis, K. A., Claypool, M. D., Li, W., Lang, W., Yam, G. C., Gelman, M. S., Ding, R., Yung, S. L., Creger, D. P., Chen, Y., Singh, R., Smuder, A. J., Wiggs, M. P., Kwon, O.-S., Sollanek, K. J., Powers, S. K., Masuda, E. S., Taylor, V. C., Payan, D. G., Kinoshita, T., Kinsella, T. M. Inhibition of Janus kinase signaling during controlled mechanical ventilation prevents ventilation-induced diaphragm dysfunction.

It has been estimated that nearly 800,000 hospital patients per year require controlled mechanical ventilation (CMV), and its use is especially prevalent in intensive care units (ICUs), where 33–40% of the patients require CMV for ∼6–7 d (1–3). Although CMV is absolutely necessary in many acute care settings, even brief periods can lead to diaphragm atrophy and weakness that can contribute significantly to delayed weaning from the ventilator (4–8). The weaning process itself can account for nearly 40% of the total time a patient spends on CMV, and a significant proportion of ventilated patients go on to experience weaning difficulty (3, 9). CMV-induced weakness of the diaphragm occurs rapidly, is progressive, and has been documented in humans and various animal models (6, 8, 10, 11). Since CMV substantially increases the cost of care, resource requirements, and medical risk for patients, strategies aimed at reducing the duration of ventilatory support would be expected to have both a clinical and cost benefit (12).

Diaphragmatic atrophy develops with surprising rapidity during CMV, as it can be observed in as little as 16–72 h in humans and 12 h in rodent models (5, 10). Despite the aggressive nature of the atrophic process under these conditions, the contractile dysfunction that develops within the diaphragm during CMV is not easily explained by atrophy alone, since isolated diaphragm strips normalized for cross-sectional area (CSA) show clear reductions in specific force (10, 11). A full understanding of the exact mechanistic chain of events that lead to CMV-induced diaphragm atrophy and contractile dysfunction remains an area of active investigation, but this process is known to critically depend on the development of mitochondrial dysfunction and increased production of reactive oxygen species (ROS) (10, 13–15). Elevated mitochondrial ROS generation is thought to trigger a proteolytic response, involving the ubiquitin–proteasome, calpain, and caspase systems, which contribute to atrophy and contractile dysfunction (10, 13–17). Why mitochondrial dysfunction occurs in the first place is unknown, but recent efforts to define signaling events associated with these changes have implicated signal transducer and activator of transcription 3 (STAT3), Fos, and FoxO as possible effectors (18).

To improve our understanding of the early signaling events that trigger CMV-induced diaphragm muscle dysfunction, we used a broad signaling assessment in diaphragm muscle fibers from mechanically ventilated rats. This effort led to the discovery of an upstream Janus kinase (JAK)/STAT3 signaling axis that triggers the accumulation of phosphorylated STAT3 within mitochondria and coincides with the mitochondrial dysfunction, muscle fiber atrophy, and decreased contractility that occur in the diaphragm during prolonged CMV.

## MATERIALS AND METHODS

Animal experiments were approved by the Institutional Animal Care and Use Committees of Rigel Pharmaceuticals, Inc., or the University of Florida and were in compliance with the Public Health Service Guidelines for the Care and Use of Laboratory Animals.

### Animals and experimental design

The JAK1/JAK3 inhibitor used in these experiments was developed as a potential therapeutic for transplant rejection, arthritis, and other systemic immune disorders in which JAK signaling plays a prominent role. Four series of experiments were conducted, and the mean mortality rate of mechanically ventilated rats was 17%. In the first 3 series of experiments, CMV studies were performed in male Sprague-Dawley rats weighing 260–280 g. Carotid artery and jugular vein cannula surgeries were performed by Harlan (Livermore, CA, USA), and the rats were shipped after a 72-h recovery period. The rats were acclimated for ∼7 d before they were randomly assigned into groups by body weight. In the first series of experiments, the rats were assigned to an unfed and conscious control group (*n*=10) or to a group mechanically ventilated for 18 h (*n*=20). In a second series of experiments, diaphragm muscles were harvested from unfed control (*n*=5–6) and mechanically ventilated rats (*n*=5–6) at different time points (1, 3, 6, and 9 h) and immediately frozen in liquid nitrogen and stored at −80°C until use in biochemical and/or molecular biology assays. In a third series of experiments, the animals were randomly assigned to 1 of 3 groups (*n*=20/group): unfed, conscious control; 18 h mechanically ventilated and treated with a Rigel JAK1/JAK3 inhibitor (1 mg/kg/h R548); or 18 h mechanically ventilated and treated with propylene glycol (vehicle for R548). Vehicle and JAK inhibitor were delivered *via* venous cannula and were infused continuously during the 18 h CMV experiment. Isoflurane was used as the anesthetic. The Institutional Animal Care and Use Committee of Rigel Pharmaceuticals, Inc., approved the experiments.

An autonomous research laboratory with a well-established preclinical model of CMV conducted a fourth series of experiments to independently determine the efficacy of JAK/STAT inhibition on CMV-induced diaphragm wasting and contractile dysfunction. Despite differences in the sex of the rats and the duration of mechanical ventilation, this model consistently leads to diaphragm atrophy and contractile dysfunction (13–15). These experiments were also undertaken to conduct mechanistic experiments, some of which required diaphragm muscles to be used immediately on dissection. Experiments were conducted in 40 female Sprague-Dawley rats (*n*=10/group; 280–300 g) randomly assigned to 1 of 4 experimental groups: 12 h anesthetized, spontaneously breathing, and unfed control, treated with propylene glycol [spontaneously breathing, vehicle treated (SB-Veh)]; 12 h anesthetized, spontaneously breathing, unfed control, treated with R548 [spontaneously breathing, R548 treated (SB-R548)] delivered at 3 mg/kg/h; 12 h of CMV treated with propylene glycol [mechanically ventilated, vehicle treated (MV-Veh)]; or 12 h of CMV treated with R548 [mechanically ventilated, R548 treated (MV-R548)] delivered at 3 mg/kg/h. Pentobarbital was used as the anesthetic. Pharmacokinetics studies had indicated that anesthetizing rats with pentobarbital results in significantly reduced plasma levels of the active form of R548, R507. Therefore, a dose of 3 mg/kg/h R548 was used. The University of Florida Institutional Animal Care and Use Committee approved the experiments.

### Mechanical ventilation

All surgical procedures were performed with aseptic techniques. In the first 3 series of experiments, tracheostomy surgeries and CMV were conducted as described previously (13–15), with slight modification. Briefly, the animals were anesthetized to a surgical plane of anesthesia with isoflurane (2–4% titrated to the animal's pain response), and a 3–5 cm longitudinal incision was made at the midline of the cervical spine. The trachea was exposed by separation of the muscles, and the trachea was then isolated from the surrounding tissues. A tracheal cannula was introduced through a small incision between the cartilage rings and secured into place by tying a suture around the trachea. The incision was closed using surgical skin staples, and the rats were maintained on CMV with isoflurane for 18 h with a volume-driven small-animal ventilator (CWE, Ardmore, PA, USA). Tidal volume was set at 0.7 ml/100 g body weight, and respiratory rate was 80 breaths/min. The carotid artery catheter was used to monitor blood pressure and to collect arterial blood samples. R548 and vehicle were delivered continuously through the jugular vein cannula. Heart rate was monitored with ECG needle electrodes, and body temperature was maintained at 37°C by a rectal temperature probe connected to a homeothermic blanket system. The animals were monitored throughout the study, and continuing care included rotating them, removing airway mucus, lubricating the eyes, and emptying the bladder as needed. Body fluid homeostasis was maintained *via* subcutaneous administration of 1.7 ml/kg body weight/2.5 h saline. To reduce airway secretions, glycopyrrolate (0.04 mg/kg) was administered subcutaneously every 2.5 h.

In the fourth series of experiments, tracheostomy surgeries and CMV were conducted as described in detail recently (13–15). Rats were ventilated with a peak-pressure ventilator, with initial tidal volume set at 1 ml/100 g and adjusted as needed, on the basis of arterial blood gas levels. At the end of the experiments, the diaphragm was collected and used immediately for contractile function studies, snap-frozen in liquid nitrogen for biochemical assays, or frozen in liquid nitrogen-cooled isopentane for histological analyses and stored at −80°C.

### Diaphragm contractile function

Diaphragm contractile function was determined by using diaphragm strips incubated *ex vivo* (10, 13). Briefly, on completion of the study, the entire diaphragm was removed and transferred to a dissecting dish containing Krebs-Henseleit physiological solution aerated with 95% O_2_–5% CO_2_ gas. A muscle strip was dissected from the midcostal region of the diaphragm, which included a portion of the central tendon and ribcage. The strip was mounted vertically with serrated jaw tissue clamps, with one end fixed to an isometric force transducer on a Tissue Organ Bath System 750 (DMT, Ann Arbor, MI, USA), and immersed in Krebs-Henseleit physiological solution aerated with 95% O_2_–5% CO_2_ gas. After a 15-min equilibration, the diaphragm strips were stimulated with a S88X Pulse Stimulator (Grass Technologies, Warwick, RI, USA) and platinum wire electrodes (Radnotti, Monrovia, CA, USA). Contractile measurements were taken at the optimal muscle length (*L*_o_), the length at which maximal force is obtained. *L*_o_ was determined by stimulating the muscle at a supramaximal voltage and systematically adjusting the muscle length. The force–frequency relationship was assessed by stimulating each strip supramaximally with 8 V pulses, with a train duration of 500 ms, at 15–160 Hz. Contractions were separated by a 2 min recovery period. Diaphragm force production was normalized as specific force based on muscle CSA. Total muscle CSA at right angles to the long axis was determined by the following calculation: total muscle CSA (mm^2^) = [muscle mass/(fiber length × 1.056)], where 1.056 is muscle density (g/cm_3_). Fiber length (cm) was measured at *L*_o_. Diaphragm contractility was assessed in all experiments, except for the time course studies.

### Gene expression

*SOCS3*, *Myf5*, and *interleukin 6* (IL-6) mRNA levels were analyzed in samples from the first experiment, by using the QuantiGene Plex 2.0 assay (Panomics/Affymetrix, Fremont, CA, USA) according to the manufacturer's instructions. Briefly, muscles were homogenized in Affymetrix proprietary homogenization buffer with proteinase K (1:60, muscle weight to volume), incubated at 65°C for 30 min, and centrifuged at 12,000 rpm for 20 min at 4°C, and the supernatant was collected for subsequent RNA quantification. Lysates where transferred to a 96-well capture plate with target-specific probe sets, and the plate was sealed and incubated overnight at 55°C for hybridization. Next, the signals of the selectively captured target RNAs were amplified *via* sequential wash and hybridization steps, followed by the addition of the streptavidin-conjugated R-phycoerythrin, which emits a fluorescent signal that is proportional to the amount of target mRNA present in the sample. mRNA concentrations were normalized to the geometric average of 4 internal control genes (*HPRT-1*, *GUSB*, *PPIA*, and *HMBS*) and expressed as median fluorescence intensity (MFI). *Atrogin-1* and muscle *RING-finger 1* (*MuRF1*) mRNA expression was measured in samples from experiment 4, with predesigned rat primer and probe sequences commercially available from Applied Biosystems (Foster City, CA, USA; ref. 14).

### Western blot analysis

Total protein extracts from frozen muscle samples were prepared by homogenizing muscle tissues in the Precellys24 Tissue Homogenizer (Bertin Corp, Rockville, MD, USA) in ice-cold RIPA buffer composed of 10 mM Tris (pH 7.2), 150 mM NaCl, 1% Triton X-100, 1% deoxycholate, 0.1% SDS, 5 mM EDTA, and complete protease/phosphatase inhibitor cocktail (Roche Diagnostics, Indianapolis, IN, USA). Tissue lysates were then centrifuged on a microcentrifuge (14,000 rpm, 20 min, 4°C; Eppendorf, Fremont, CA, USA), the protein content of the supernatant (total muscle extract) was determined with a Micro-BCA protein estimation kit (Pierce, Rockford, IL, USA), and the samples were subjected to protein normalization. The samples were then boiled in 4× NuPAGE sample buffer containing LDS/DTT, and equal aliquots (10–20 μg protein extract) were subjected to SDS-PAGE separation on premade 4–12% Bis-Tris gradient gels, with MOPS/SDS used as a running buffer system, per the manufacturer's recommendations (Invitrogen-Life Technologies, Carlsbad, CA, USA). Proteins electroblotted onto PVDF membranes were incubated with the following primary antibodies and the appropriate secondary antibodies: phospho-STAT3 [9131; Tyr705; Cell Signaling Technology (CST), Danvers, MA, USA], STAT3 (9132; CST), pSTAT3-S727 (NB2-12965; Novus Biologicals, Littleton, CO, USA), phospho-p38 (9215; Thr180/Tyr182; CST), myogenin (ab124800; Abcam, Cambridge, MA, USA), Ac-Histone H3 (9649; CST), tubulin (5346; CST), GRIM-19 [sc-136431; Santa Cruz Biotechnology (SCB), Dallas, TX, USA], 4-hydroxynonenal (4-HNE; ab46545; Abcam), lactate dehydrogenase A (LDHA; 2012S; CST), proliferating cell nuclear antigen (PCNA; sc-56; SCB), voltage-dependent anion channel (VDAC; 4661; CST), cytochrome *c* (4272; CST), caspase-3 (9664; CST), calpain 1 (2556; CST), and α-spectrin (sc-48382; SCB). Atrogin-1 (AP2041) and MuRF1 (MP3401) were from ECM Biosciences (Versailles, KY, USA). Blots were developed with an ECL Plus enhanced-chemiluminescence reagent kit obtained from Amersham Pharmacia Biotech (Piscataway, NJ, USA) and detected on Kodak Bio-MAX MR film (VWR Scientific, San Francisco, CA, USA). Tubulin was used as a loading control, to confirm standardized protein loading and transfer. The bands were analyzed using the public domain ImageJ program (U.S. National Institutes of Health, Bethesda, MD, USA; http://rsb.info.nih.gov/ij/index.html), quantified by densitometry, and normalized to the appropriate loading controls.

### Protein carbonyl levels

Protein carbonyl levels were assessed in diaphragm muscle by using the OxiSelect Protein Carbonyl ELISA Kit (Cell Biolabs, San Diego, CA, USA), according to the manufacturer's instructions.

### IL-6 levels

Plasma IL-6 levels were determined in samples from the third series of experiments, by using the Luminex Rat Cytokine Kit (R&D Systems, Minneapolis, MN, USA), according to the manufacturer's instructions. Briefly, whole blood was collected in K_2_ EDTA tubes and centrifuged at 4000 rpm for 5 min at 4°C, and the supernatant (plasma) was collected and stored at −20°C for subsequent analysis. Plasma samples were incubated with a cocktail of beads coated with capture antibodies to specific cytokines and washed several times. A secondary biotinylated detection antibody that bound to the cytokine captured on the bead was incubated with the samples. The reaction mixture was then incubated with streptavidin-penicillin conjugate to complete the reaction on the surface of the microbead. The microbeads were then passed through a laser that excites the internal dyes marking the microbead set, a second laser that excites the reporter molecule on the surface of the bead, and high-speed digital signal processor that identify each microbead and quantify the results.

Diaphragm and lung IL-6 levels were assessed in samples from the third series of experiments with an IL-6 immunoassay kit (R&D Systems). Briefly, samples were homogenized in RIPA buffer containing PhosStop Phosphatase Inhibitor Cocktail (Roche Diagnostics) and homogenization at 4°C in a PreCellys 24 Bead Homogenizer (Bertin Corp.), according to the manufacturer's instructions. The samples were then centrifuged at 13,000 *g* for 20 min to remove nonsoluble proteins. The supernatant was collected, assessed for protein concentration, and standardized.

### Myofiber CSA

Diaphragm strips from the third series of experiments were used for histologic assessment of CSA. Because diaphragm samples from that series of experiments were damaged during cold storage and subsequent tissue processing, CSA was instead assessed in samples from a previous study that used a related JAK inhibitor from the same chemical scaffold series (R545). In that study, STAT3 phosphorylation, contractile dysfunction, and rescue by treatment with the JAK inhibitor (R545) were nearly identical to results in the studies of R548. Samples from this experiment were frozen in isopentane cooled by liquid nitrogen and stored at −80°C. Frozen tissue was sectioned at 6 μm and mounted on slides, fixed in pure acetone at −20°C for 10 min, and stained with anti-laminin antibody (L9393; Sigma-Aldrich, St. Louis, MO, USA) at 1:200 for 30 min in PBS with 5% BSA. Alexa Fluor 568 anti-rabbit secondary antibody (A11011; Invitrogen, Grand Island, NY, USA) was applied at 1:200 for 30 min. Slides were digitally imaged with a ×4 objective to capture each entire tissue section with an IXMicro fluorescence microscope (Molecular Devices, Sunnyvale, CA, USA).

Image analysis was performed with CellProfiler (http://cellprofiler.org). Images were analyzed separately, not as a single montage of the entire section. Thresholding was performed by using Otsu's method, minimizing weighted variance with 3 classes. The middle class was assigned to background. Morphologic techniques were applied to provide outlines for each fiber. Images with poor or incomplete thresholding were discarded from the analysis by manual inspection. Multiple image fields were usually necessary for complete coverage of the tissue sections. All image results for each section were summed, and the results for each section were averaged across sections for each animal. Muscle fiber size is reported in square micrometers.

### Preparation of permeabilized muscle fibers

Samples from the fourth study were permeabilized as reported recently (10, 19). Briefly, small portions (∼25 mg) of diaphragm muscle were dissected and placed on a plastic Petri dish containing ice-cold buffer X (60 mM K-MES, 35 mM KCl, 7.23 mM K_2_EGTA, 2.77 mM CaK_2_EGTA, 20 mM imidazole, 0.5 mM DTT, 20 mM taurine, 5.7 mM ATP, 15 mM PCr, and 6.56 mM MgCl_2_, pH 7.1). The muscle was trimmed of connective tissue and cut down to fiber bundles (4–8 mg wet weight). With the aid of a microscope and using a pair of extra-sharp forceps, we separated the muscle fibers gently in ice-cold buffer X, to maximize the surface area of the fiber bundle. To permeabilize the myofibers, we incubated each fiber bundle in ice-cold buffer X containing 50 μg/ml saponin on a rotator for 30 min at 4°C. The permeabilized bundles were then washed in buffer Z (110 mM K-MES, 35 mM KCl, 1 mM EGTA, 5 mM K_2_HPO4, 3 mM MgCl_2_, 0.005 mM glutamate, 0.02 mM malate, and 0.5 mg/ml BSA, pH 7.1).

### Mitochondrial respiration in permeabilized fibers

Respiration was measured in samples from the fourth study polarographically in a respiration chamber maintained at 37°C (Hansatech Instruments, Norfolk, UK; refs. 10, 19). After the respiration chamber was calibrated, permeabilized fiber bundles were incubated with 1 ml of respiration buffer Z containing 20 mM creatine to saturate creatine kinase. Flux through complex I was measured by using 5 mM pyruvate and 2 mM malate. ADP-stimulated respiration (state 3) was initiated by adding 0.25 mM ADP to the respiration chamber. Basal respiration (state 4) was determined in the presence of 10 μg/ml oligomycin, to inhibit ATP synthesis. The respiratory control ratio (RCR) was calculated by dividing state 3 by state 4 respirations.

### Preparation of mitochondrial fraction from frozen diaphragm

All buffer reagents used for preparing mitochondria isolation buffer (250 mM sucrose, 5 mM Tris, and 2 mM EGTA; pH to 7.4 at 4°C) and washing buffer were from Sigma-Aldrich. Mitochondria washing buffer is essentially the isolation buffer, but contains protease inhibitor cocktail (cat. no. 05892791001) and phosphatase inhibitor cocktail (cat. no. 04906837001; both from Roche Diagnostics). RIPA buffer (cat. no. R3792) from Teknova (Hollister, CA, USA), containing protease and phosphatase inhibitor cocktail, was used to dissolve the mitochondria pellet. All protein gels, PVDF membranes, and the Western blot system were obtained from Life Technologies, unless otherwise specified. For mitochondrial fractionation, frozen rat diaphragm tissues from the fourth series of experiments (∼100 mg wet weight) were placed in 2 ml tissue-homogenizing tubes containing 1 ml mitochondria isolation buffer and porcelain beads (2.8 mm diameter; cat. no. G-3290-22; Bioexpress, Kaysville, UT, USA). The tissues were then homogenized carefully in a Precellys24 homogenizer (Bertin Corp.) at 4°C and 6000 rpm, programmed for 3 × 15 s spin with 20 s intermissions. Tissue homogenates were then immediately placed on ice for 10 min before low-speed centrifugation (2000 *g* for 5 min at 4°C) on a tabletop refrigerated microcentrifuge (model 5417R; Eppendorf) to remove large particles. Low-speed centrifugation was repeated 2 times, and the clear supernatant obtained was then subjected to medium centrifugation (12,000 *g* for 15 min at 4°C). Both pellet (mitochondria) and the supernatant were separated carefully, and the pellet was washed ≥2 times with the washing buffer containing protease and phosphatase inhibitors, to ensure its purity. Finally, the mitochondrial pellet was dissolved in 100 to 150 μl RIPA buffer containing protease and phosphatase inhibitors. For Western blot analysis, aliquots from both the supernatant and the pellet were normalized for protein concentration by Bradford BCA assay and were loaded equally onto 4–12% Bis-Tris gels, followed by protein blotting onto PVDF membranes. VDAC served as a marker for mitochondria, and LDHA and PCNA served as markers for cytosol and nuclear fraction, respectively.

### Statistical analysis

Results are reported as means ± sem. Statistical analysis was performed by using Student's *t* test, when the mean results from 2 groups were compared, or by ANOVA followed by Tukey's *post hoc* test, when the mean results from >2 groups were compared. Values of *P* < 0.05 were considered statistically significant.

## RESULTS

### Mechanical ventilation leads to robust phosphorylation of STAT3 on Tyr705 in the diaphragm

**Figure 1.**
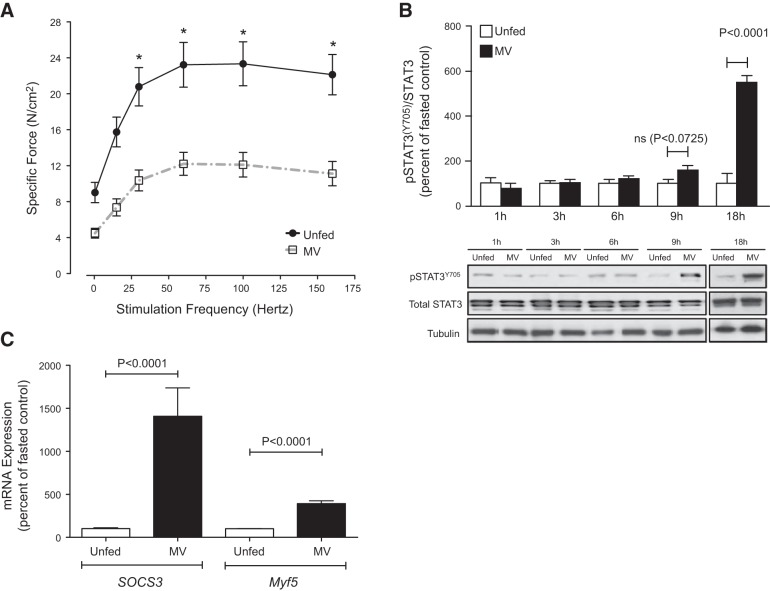
CMV results in activated phosphorylation of STAT3 on Tyr705 and diaphragm contractile dysfunction. Diaphragms from mechanically ventilated (MV) rats and unfed controls were analyzed. *A*) *Ex vivo* force–frequency relationship from mechanically ventilated (*n*=13) rats and unfed controls (*n*=7). *B*) Western blots for total STAT3 and phospho-STAT3^Y705^ from a representative set of mechanically ventilated rats and unfed controls (1, 3, 6, and 9 h, *n*=5–6 rats/group; 18 h, *n*=6–8). *C*) Messenger RNA levels of the STAT3 downstream target genes *SOCS3* and *Myf5* from mechanically ventilated (*n*=9) rats and unfed controls (*n*=10). Mechanical ventilation period of 18 h. Results are means ± sem. *P < 0.05; Student's *t* test.

Given the links between STAT3, muscle wasting (20), and factors that might influence diaphragm dysfunction during CMV (18), we first focused on whether STAT3 was activated in the diaphragm of tracheostomized 12-week-old male Sprague-Dawley rats subjected to 18 h of CMV. Since mechanically ventilated rats do not receive nutritional supplementation during the ventilation period, controls consisted of conscious, nonventilated rats that were denied access to food for the duration of the experiment. CMV significantly reduced the specific force (*P*<0.05; **Fig. 1*A***) and markedly increased activating phosphorylation of STAT3 (Tyr705) in the diaphragms of ventilated rats relative to nonventilated unfed controls (Fig. 1*B*). In addition, mRNA levels of downstream targets of JAK/STAT3 activation, such as *SOCS3* and *Myf5*, were robustly upregulated in the diaphragm in response to CMV, lending further support to the presence of activated STAT3 in this muscle (Fig. 1*C*). We next examined whether the levels of total and phosphorylated STAT3 were altered at earlier time points after initiation of CMV. Diaphragm muscles were studied between 1 and 9 h after mechanical ventilation. Total STAT3 levels were not altered at any time point. STAT3 phosphorylation was unchanged after 1, 3, and 6 h, but tended to be increased after 9 h of CMV (*P*=0.0725; Fig. 1*B*).

### Continuous i.v. infusion of a selective JAK1/JAK3 inhibitor prevents CMV-induced induction of phospho-STAT3Y705 and attenuates the development of diaphragm contractile dysfunction, atrophy, and mitochondrial dysfunction

**Figure 2.**
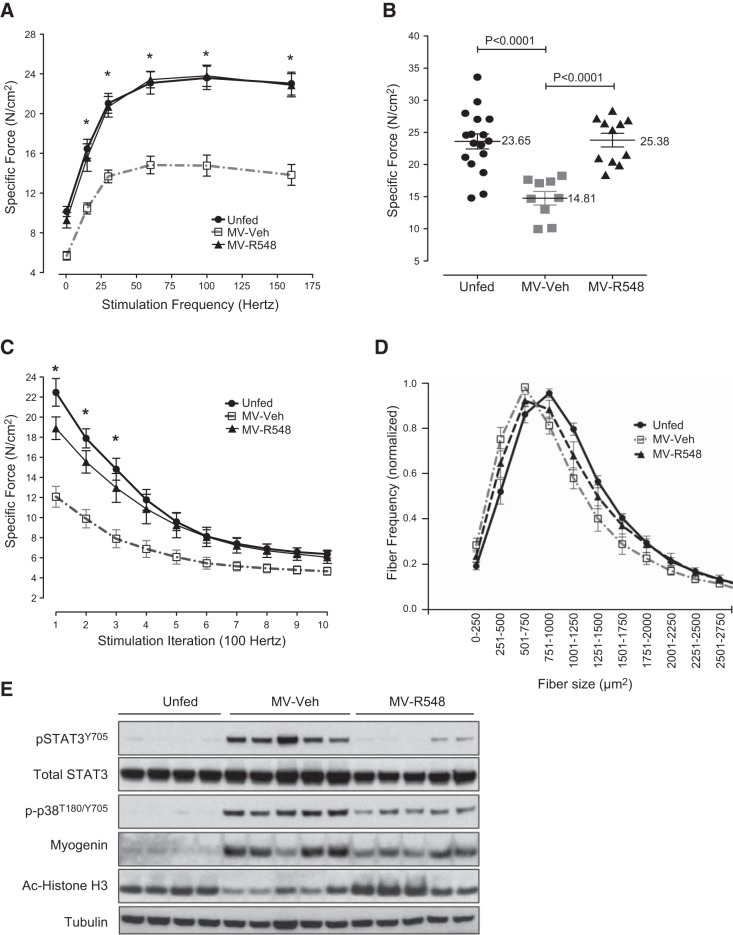
Treatment with a JAK1/JAK3 inhibitor prevents controlled mechanical VIDD and atrophy. Diaphragms from unfed control rats (*n*=17), MV-R548 rats (*n*=11), or MV-Veh rats (*n*=9) were analyzed. *A*) Diaphragm strip *ex vivo* force–frequency relationship. *P < 0.05, control and MV-R548 *vs.* MV-Veh; 2-way ANOVA. *B*, *C*) Specific force generation at 100 Hz (*B*) and (*C*) fatigue development after 18 h of CMV. *P < 0.05, control *vs*. MV-Veh; 2-way ANOVA. *D*) Muscle fiber CSA from a representative set of unfed control rats (*n*=9), MV-R548 rats (*n*=7), or MV-Veh rats (*n*=8). *E*) Representative Western blot from unfed control, MV-R548, and MV-Veh rats. Mechanical ventilation period of 18 h. Results are means ± sem.

**Figure 3.**
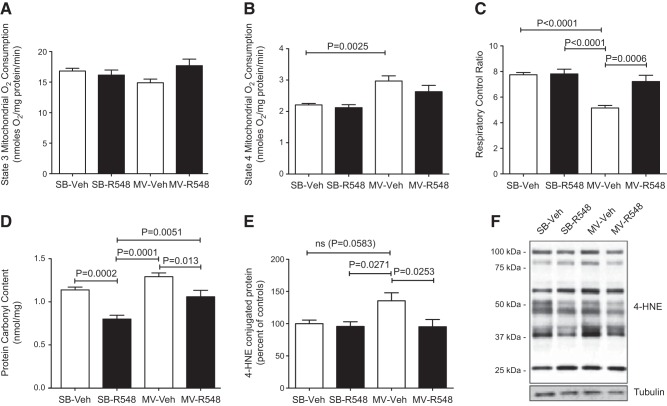
Treatment with a JAK1/JAK3 inhibitor prevents CMV-induced mitochondrial dysfunction and oxidative damage. *A–C*) State 3 (*A*), State 4 (*B*), and RCR (*C*) in saponin-permeabilized diaphragm fibers of SB-Veh, SB-R548, MV-Veh, and MV-R548 rats. *D*, *E*) Protein carbonyl content (*D*) and 4-HNE-conjugated proteins (*E*) in total diaphragm homogenates. *F*) Representative blot for 4-HNE. CMV period of 12 h. Values are means ± sem (*n*=10/group). *P* values calculated by 1-way ANOVA with Tukey's *post hoc* analysis.

**Figure 4.**
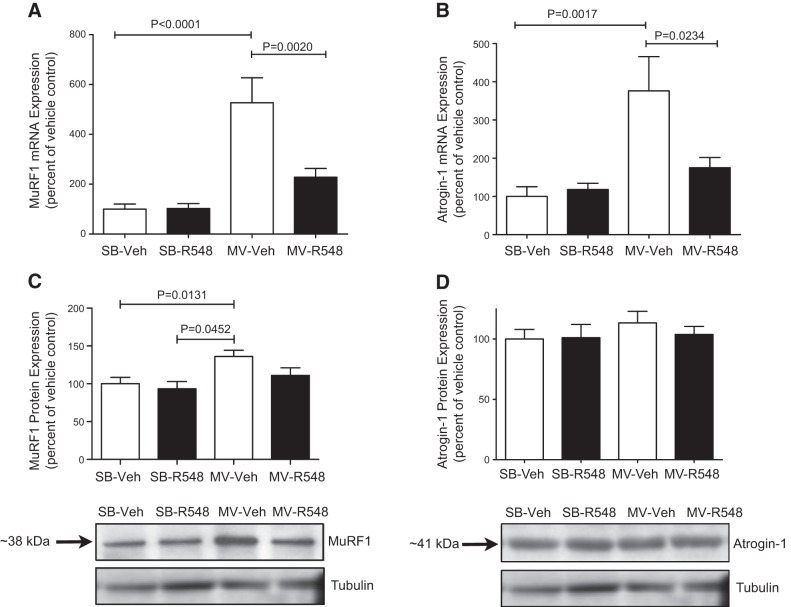
Treatment with a JAK1/JAK3 inhibitor prevents CMV-induced mRNA up-regulation of atrophic E3 ubiquitin ligases. Messenger RNA levels of E3 ubiquitin ligases *MuRF1* (*A*) and *atrogin-1* (*B*) mRNA levels and protein levels of MuRF1 (*C*) and atrogin-1 (*D*) in the diaphragms of SB-Veh, SB-R548, MV-Veh, and MV-R548 rats. CMV period of 12 h. Values are means ± sem (*n*=10/group). *P* values calculated by 1-way ANOVA with Tukey's *post hoc* analysis.

Having demonstrated robust elevations in STAT3 activation during CMV, we hypothesized that signals transmitted through the JAK/STAT pathway are responsible for the development of diaphragm atrophy and dysfunction. To test this hypothesis directly, a second series of experiments was conducted in mechanically ventilated rats continuously infused with 1 mg/kg/h R548, the prodrug of a selective JAK1/JAK3 inhibitor, R507 (Supplemental Fig. S1 and ref. 21), for the duration of the ventilation period and then assessed for both contractile dysfunction and atrophy. The end point (18 h) plasma exposure to R507, the active prodrug cleavage product, was ∼712 ng/ml (data not shown). R548 treatment led to nearly complete rescue of diaphragm force-generating capacity (median rescue of 100% relative to untreated ventilated controls at 100 Hz; *P*=0.0143), as observed in either force–frequency or fatigue profiling of isolated diaphragm strips (**Fig. 2*A–C***). Similar results were obtained with the JAK 1/3 inhibitor CP690550 (22) infused at 3 mg/kg/h (data not shown). Further, 18 h of CMV resulted in a 25% reduction in diaphragm fiber CSA, and inhibition of JAK signaling prevented nearly half of this loss, suggesting that at least part of its benefit is attributable to the prevention of atrophic processes (Fig. 2*D*). As expected, R548-mediated rescue was accompanied by significant reductions in phospho-STAT3^Y705^ levels within the diaphragm (Fig. 2*E*). A broad screening of diaphragm from mechanically ventilated rats, using Western blots, identified several additional proteins with notable induction patterns that were also modulated by treatment with R548, indicating that these were positioned downstream of JAK signaling and could contribute to diaphragm dysfunction (Fig. 2*E*). For example, partial attenuation of phosphorylated p38 (Thr180/Ser182) and myogenin expression was also observed, although these were reduced to a lesser extent than phospho-STAT3^Y705^, suggesting that at least some portion of the activation of these proteins lay downstream of JAK function (Fig. 2*E*). Since both myogenin expression and muscle atrophy have been linked to changes in the activity of HDAC proteins (23), we assessed the level of histone H3 acetylation as a downstream marker of HDAC function. CMV markedly decreased the levels of acetylated histone H3 in the diaphragm, and R548 treatment fully reversed these changes, suggesting a role for HDACs downstream of JAK/STAT3 signaling events (Fig. 2*E*). Since mitochondrial damage is thought to play a central role in the development of ventilator-induced diaphragm dysfunction (VIDD; ref. 10), a fourth series of experiments was carried out to assess the impact of R548-mediated JAK inhibition on mitochondrial function by comparing the respiratory control ratios of mitochondria from intact diaphragm fibers in R548-treated *vs.* untreated rats subjected to 12 h of CMV. Consistent with earlier results, JAK inhibition fully prevented contractile dysfunction (Supplemental Fig. S2), accompanied by rescue of impaired coupling within mitochondria (**Fig. 3*A***–***C***) and reductions in the level of ROS-mediated protein carbonyls (Fig. 3*D*) and 4-HNE-conjugated proteins (Fig. 3*E*), indicating that JAK inhibition reduces mitochondrial dysfunction and oxidative stress as part of its protective mechanism.

### JAK1/JAK3 inhibition during mechanical ventilation reduces diaphragmatic protease activation and proteolysis

**Figure 5.**
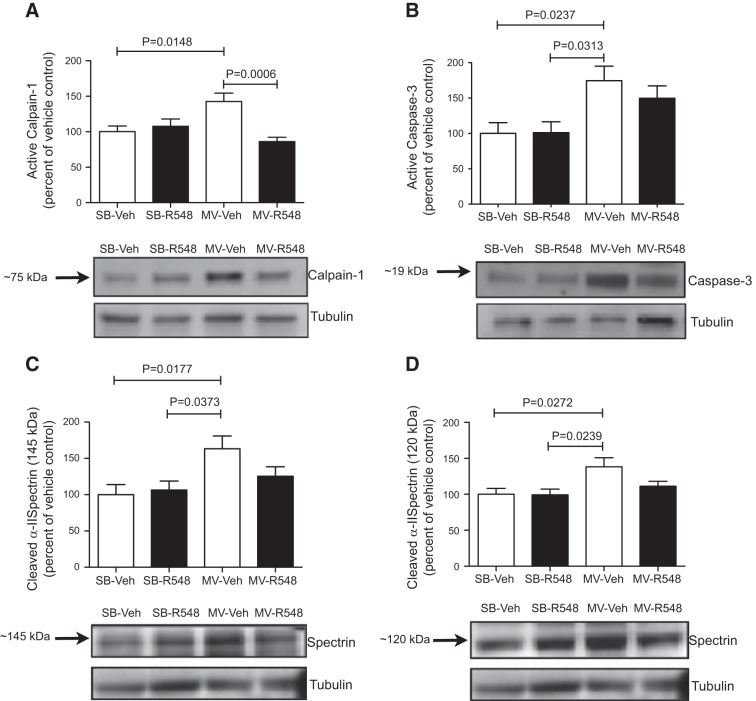
Treatment with a JAK1/JAK3 inhibitor attenuates activation of key proteases during CMV. Protein levels of active calpain-1 (*A*), active caspase-3 (*B*), calpain-cleaved 145 kDa α-II spectrin breakdown product (*C*), and 120 kDa caspase-3-cleaved α-II spectrin breakdown product (*D*) in the diaphragms of SB-Veh, SB-R548, MV-Veh, and MV-R548 rats. Mechanical ventilation period of 12 h. Values are means ± sem (*n*=10/group). *P* values calculated by 1-way ANOVA with Tukey's *post hoc* analysis.

*C*MV-induced oxidative stress can activate proteases in the diaphragm, which, in turn, contributes to diaphragm wasting and contractile dysfunction (10, 13, 15, 24). Because JAK inhibition prevented diaphragmatic mitochondrial dysfunction and oxidative stress, we next tested in samples from the fourth study whether JAK signaling may be involved in regulating key proteolytic pathways during CMV. JAK inhibition prevented CMV-induced increases in the mRNA expression of the E3 ubiquitin ligases *atrogin-1* and *MuRF1* in the diaphragm (**Fig. 4*A*, *B***). Whereas CMV did not influence atrogin-1 protein levels, MuRF1 protein levels were significantly increased in diaphragm muscles from CMV rats, and treatment with R548 successfully blocked this increase (Fig. 4*C*, *D*). CMV markedly increased the amount of active calpain-1 and caspase-3 (**Fig. 5*A*, *B***), and this was accompanied by elevated levels of calpain-specific (145 kDa) and caspase-3-specific (120 kDa) spectrin breakdown products (Fig. 5*C*, *D*). Treatment with R548 partially attenuated protease activation and the accumulation of calpain- and caspase-3-cleaved spectrin (Fig. 5*A–D*). Collectively, these data suggest that JAK inhibition during CMV protected diaphragm from atrophy and dysfunction by blunting activation of the ubiquitin–proteasome, calpain, and caspase proteolytic systems.

### Mechanical ventilation leads to increased accumulation of Ser705- and Tyr705-phosphorylated STAT3 within mitochondria, effects prevented by JAK inhibition

**Figure 6.**
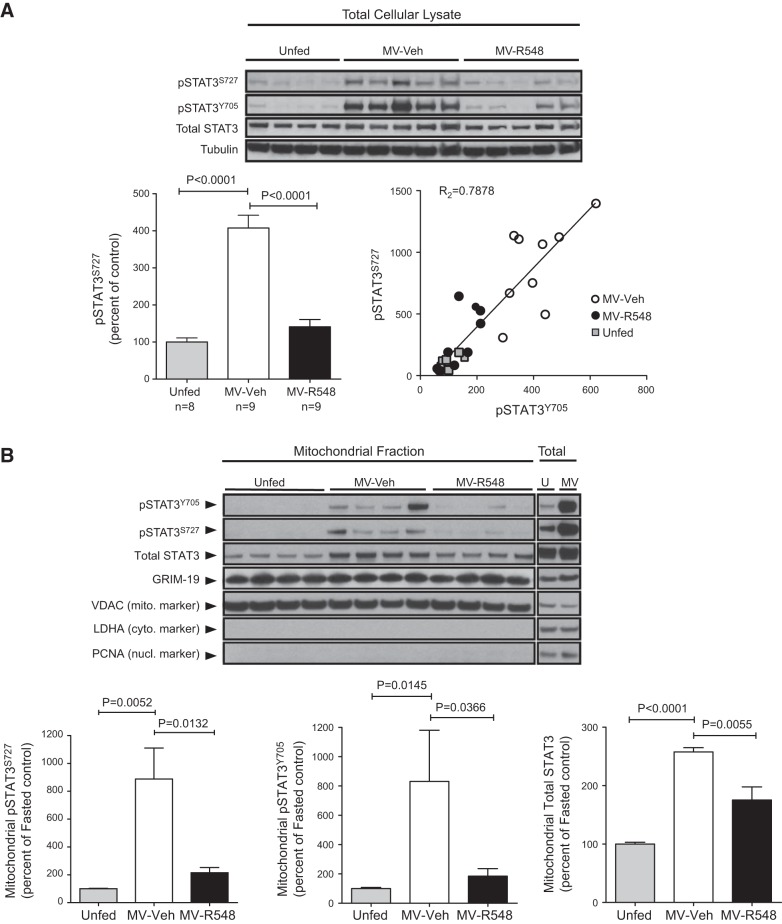
CMV increases Ser705 phosphorylation and mitochondrial accumulation of phospho-STAT3 (Ser727 and/or Tyr705/Ser727) within diaphragm muscle. *A*) Diaphragms from unfed control rats (*n*=8), MV-R548 rats (*n*=9), or MV-Veh rats (*n*=9) were analyzed by Western blot of total muscle lysate (representative animals depicted in blot) and quantitation. Correlation between increases in phospho-STAT3^S727^ and phospho-STAT3^Y705^ were determined. *B*) Purified mitochondrial fractions (mito) and total lysates (total) were subjected to Western blot analysis for pSTAT3^S727^, pSTAT3^Y705^, total STAT3, GRIM-19, VDAC (mitochondrial marker), LDHA (cytoplasmic marker) and PCNA (nuclear marker). Unfed (U) controls, *n* = 10; MV-Veh (MV), *n* = 6; MV-R548, *n* = 7. Results are means ± sem. Mechanical ventilation period of 18 h. *P* values calculated by 1-way ANOVA with Tukey's *post hoc* analysis.

Although STAT3 is best characterized by its role as a nuclear transcription factor, recent findings have demonstrated that Ser705-phosphorylated STAT3 is imported into mitochondria, where it associates with complex I of the electron transport chain (ETC) and modulates respiration (25–27). Since this association can lead to ROS production in certain contexts (28, 29) and ROS-related mechanisms are thought to play a prominent role in the development of VIDD, we analyzed in samples from the second series of experiments the levels of Ser705-phosphorylated STAT3 in total cellular lysates. As shown in **Fig. 6*A***, CMV led to significant increases in the amount of phospho-STAT3^S727^, and this effect was prevented by inhibition of JAK signaling, indicating that these events were coupled, albeit indirectly, since JAKs are tyrosine kinases and are incapable of phosphorylating this site directly. Notably, the increased abundance of phospho-STAT3 (Ser705 and/or Tyr705) induced by CMV was accompanied by substantial increases in its accumulation within the mitochondria; this effect was also prevented by treatment with a JAK inhibitor (Fig. 6*B*). GRIM-19, a component of complex I, is critical for the import of phospho-STAT3^S727^ into mitochondria, and, similar to STAT3, its presence there has also been linked to increased ROS generation (28). GRIM-19 was readily detected within mitochondria, but CMV did not lead to additional increases of this protein (Fig. 6*B*).

### Mechanical ventilation leads to increased circulating IL-6, but does not alter diaphragm or lung IL-6 protein expression

**Figure 7.**
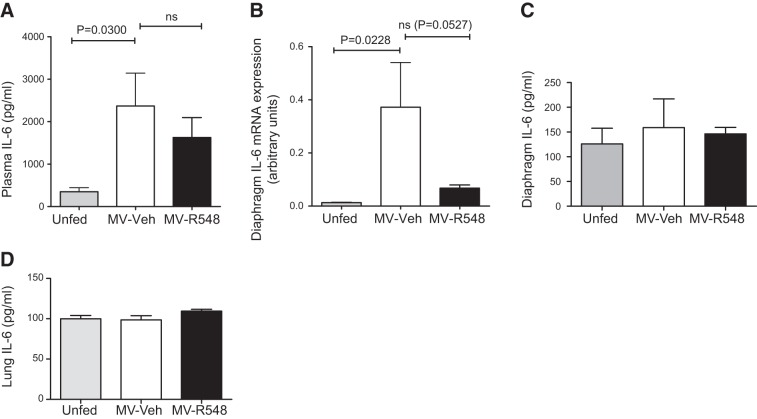
CMV increases plasma IL-6. A) Plasma IL-6 levels in unfed control (*n*=8) MV-Veh (*n*=6), and MV-R548 (*n*=10) rats. *B*) Messenger RNA levels of *IL-6* in diaphragm of control (*n*=10), MV-Veh (*n*=9), and MV-R548 (*n*=10) rats. *C*, *D*) Protein levels of IL-6 in diaphragm (*C*) and lung (*D*) of control (*n*=8), MV-Veh (*n*=7), and MV-R548 (*n*=9) rats. CMV period of 18 h. Values are means ± sem. *P* values calculated by 1-way ANOVA with Tukey's *post hoc* analysis.

IL-6 is a well characterized activator of JAK/STAT3 signaling, can be produced by muscle (30) or lung (31) in response to physical damage, and has been associated with muscle atrophy (20). We therefore explored the role of this cytokine during CMV. Plasma levels of IL-6 were significantly increased during CMV, as was *IL-6* gene expression in the diaphragm (**Fig. 7*A*, *B***). However, IL-6 protein levels in the diaphragm were unchanged by mechanical ventilation (Fig. 7*C*), suggesting that an autocrine IL-6 signaling loop within the diaphragm does not contribute to elevated levels of plasma IL-6. Previously, ventilation-induced lung damage has been shown to result in IL-6 up-regulation locally within injured lung tissue, and we therefore investigated whether this mechanism also contributes to the observed increases in circulating IL-6 plasma levels. These experiments demonstrated that IL-6 protein levels were not increased in the lungs after 18 h of CMV, suggesting that ventilation-induced lung damage is unlikely to contribute to an increase in circulating IL-6 (Fig. 7*D*).

## DISCUSSION

Despite the life-saving role played by CMV in modern medicine, one unintended consequence of its use is the rapid and progressive induction of contractile dysfunction and atrophy of the diaphragm. This CMV-induced diaphragmatic weakness is clinically important because it can substantially delay the weaning process and thereby significantly contribute to the increased cost of care, resource requirements, and medical risk for some patients who might otherwise be incrementally closer to recovery (1–12). However, there are currently no therapeutic agents available in the clinic that are capable of preventing the development of VIDD. Understanding how diaphragm weakness develops in this context may offer new avenues for therapeutic intervention.

The results described in this article and elsewhere (10) suggest a mechanistic chain of events within the diaphragm during VIDD, in which mitochondrial dysfunction and the generation of ROS occur upstream of, and are causally linked to, diaphragm atrophy and decreases in diaphragm force-generating capacity. In recent years, much progress has been made in defining the events that occur downstream of mitochondrial dysfunction, but little is known about how and why these pathologic changes are triggered in the first place. The findings presented in this article expand the molecular understanding of how VIDD develops, by identifying JAK signaling as a critical triggering mechanism upstream of STAT3 phosphorylation, mitochondrial dysfunction, ROS production, diaphragm fiber atrophy, and contractile dysfunction. These conclusions are based primarily on the ability of a selective JAK1/JAK3 inhibitor (21) to rescue diaphragm force-generating capacity, preserve muscle mass, and prevent decline of mitochondrial function during CMV.

We focused our efforts on JAK/STAT3 signaling for 2 reasons. First, changes in STAT3 protein levels within the diaphragm have been linked to prolonged CMV in humans (18), although the central nature of its role in driving diaphragm dysfunction had not been fully established. Second, there is evidence that mitochondrially translocated STAT3 can influence ROS generation through modulation of ETC function (25–27). Mitochondrial STAT3 has been linked directly to increased ROS generation in at least two distinct signaling contexts: TNF-α-mediated necroptosis in L929 mouse fibrosarcoma cells (28) and NGF-dependent neurite outgrowth in PC12 cells (29). In addition, increased superoxide generation in vascular smooth muscle cells exposed to angiotensin II is prevented by JAK/STAT inhibition (30). It is worth noting that the consequences of increased mitochondrial STAT3 appear to be tissue and/or context dependent. For example, in contrast to the increased ROS production associated with TNF-α or NGF signaling, as described above, mitochondria-targeted STAT3 appears to reduce ROS generation in several cell types subjected to hypoxia, but has no impact in these same cells under conditions of normoxia (31, 32).

The activation of STAT3 in the diaphragm during CMV was not limited to the canonical JAK target site at Tyr705, as robust increases in phosphorylation of Ser727 also occurred. These changes during CMV were associated with increased mitochondrial translocation of phosphorylated STAT3, suggesting a mechanistic link between activation of this pathway and the observed mitochondrial dysfunction. The import of STAT3 into mitochondria is mediated by its association with GRIM-19, a component of complex I, and depends critically on STAT3 Ser727 phosphorylation (25, 26). This process can occur independent of phosphorylation of STAT3 on Tyr705, the direct JAK target site, and does not require a functional DNA binding domain. Since phosphorylation of STAT3 on Ser727 appears to control its access to mitochondria, the significant levels of Tyr705-phosphorylated STAT3 that we observed in mitochondrial fractions is a likely indicator that the dominant species under these conditions is modified at both sites.

In addition to the expected block of Tyr705 phosphorylation, somewhat surprisingly, inhibition of JAK signaling also prevented phosphorylation of STAT3 on Ser727. This finding indicates that these two different phosphorylation events are coupled to JAK signaling and that JAK sits upstream of the serine kinases responsible for modulating STAT3. Consistent with a gatekeeping role for Ser727 in mitochondrial import, this blockade largely prevented the ventilation-induced increases in both forms of phospho-STAT3 within mitochondria, and, notably, this effect was accompanied by a normalization of mitochondrial respiration control ratios, reductions in markers of oxidative damage such as 4-HNE-conjugated proteins, and the prevention of diaphragm contractile dysfunction. These data provide important and novel insights regarding the upstream mechanisms involved in CMV-mediated oxidative stress and diaphragm weakness, by showing for the first time the involvement of JAK signaling in muscle contractile dysfunction. It is not completely understood how oxidants reduce diaphragm specific force production, but it may occur through post-translational protein modifications, such as oxidation of myofibrillar proteins and/or altered phosphorylation of myofibrillar proteins *via* oxidant sensitive kinases or phosphatases (see ref. 33 for review). Oxidant-mediated activation of proteases (13) and/or oxidative damage to myofibrillar proteins, which renders them susceptible to proteolytic degradation (17), may also contribute to diaphragm weakening during CMV. Muscle contractility is sensitive to reduction-oxidation (redox) balance, such that elevated levels of oxidants can suppress muscle-specific force (see ref. 33 for review), and there is now compelling evidence that oxidative stress plays a major role in VIDD (10, 13, 14, 15). Mitochondrial dysfunction appears to contribute significantly to oxidative stress during CMV, as it was recently reported that treatment with a mitochondria-targeted antioxidant during CMV prevented mitochondrial respiratory dysfunction, elevated mitochondrial ROS production, and oxidative damage in diaphragm muscle (10). Notably, the mitochondria-specific antioxidant also prevented diaphragm contractile dysfunction (10), thereby connecting mitochondrial defects and elevated mitochondrial ROS production to diminished diaphragm-specific force.

In addition to the qualitative changes that reduce the diaphragm's ability to contract after CMV (*i.e*., contractile strength is significantly reduced, even after normalizing for CSA), the absolute loss of muscle mass (atrophy) associated with CMV is also a likely contributor to the overall weakness that develops within the diaphragm. In the present study, inhibition of JAK/STAT3 signaling prevented approximately half of the atrophy associated with CMV, supporting the concept that muscle wasting during CMV is due, at least in part, to JAK signaling. Improved diaphragm mass may have been due to diminished activation of MuRF1, since CMV-mediated up-regulation of MuRF1 mRNA and protein levels were reduced by JAK/STAT3 inhibition. MuRF1 is an E3 ubiquitin ligase that marks specific contractile proteins for selective degradation by proteasome-dependent mechanisms during muscle atrophy and plays a key role in muscle wasting (34–36). Although the precise mechanisms cannot be determined from these experiments, JAK signaling may act *via* p38 and/or myogenin, since these molecules can regulate MuRF1 expression (37, 38), and JAK inhibition reduces MV-mediated changes in these pathways. However, in contrast to findings in a recent report (10), atrogin-1 protein levels were unaltered by CMV in the present study and therefore are unlikely to contribute to CMV-induced atrophy. Of interest, JAK-inhibition also blunted activation of the calpain and caspase-3 proteolytic systems, which have been suggested to contribute to CMV-mediated diaphragm atrophy and contractile dysfunction (10, 13, 16, 17).

Reflective of its diverse roles in normal biological and pathophysiological processes, numerous serine kinases signal through STAT3 *via* Ser727 phosphorylation, including mTOR (39), PKC (40), RIPK-1(28), and the 3 major MAPK pathways: ERK, p38, and JNK (41, 42). The components of the signaling pathway that lie between JAK and STAT3^S727^ are unknown; however, mTOR phosphorylation (activation) is unchanged during CMV (data not shown), likely ruling out its involvement in STAT3^S727^ phosphorylation. CMV-induced activation (phosphorylation) of p38 was blunted by JAK inhibition, suggesting that p38 accounts for at least some portion of phospho-STAT3^S727^ up-regulation.

It is not known from these experiments precisely how the JAK/STAT3 signaling pathway becomes activated during VIDD. A wide variety of molecules act through JAK/STAT3 signaling, and we found that plasma levels of one of these ligands, IL-6, were elevated during CMV. Increased *IL-6* mRNA expression in the diaphragm during CMV suggests that an autocrine loop contributes to the activation of STAT3. However, STAT3 activation *via* autocrine signaling is unlikely, since protein levels of IL-6 in the diaphragm are unaltered by mechanical ventilation. Alternatively, intrinsic activation of JAK signaling in response to oxidative stress has been reported in a variety of cell types (43), but since JAK inhibition prevents oxidative stress during CMV, its activation most likely occurs upstream of this event. However, it is still possible that such intrinsic JAK/STAT3 activation mechanisms function in self-amplification loops during later stages when mitochondrial dysfunction and ROS production have already progressed.

**Figure 8.**
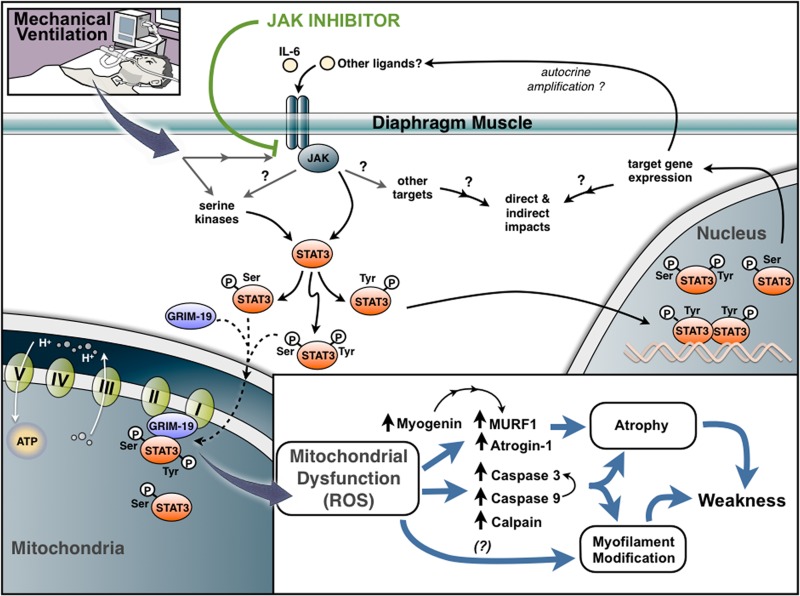
Proposed model for role of JAK signaling in VIDD. JAK signaling is activated by CMV and functions as a critical triggering mechanism upstream of STAT3 phosphorylation (Tyr705 and Ser705), mitochondrial dysfunction, ROS production, atrophy, and muscle weakness. Mechanical ventilation results in the mitochondrial accumulation of phospho-STAT3 (singly phosphorylated on Ser727 or doubly phosphorylated at both Ser727 and Tyr705). Import into mitochondria is facilitated through interaction of phospho-STAT3 with GRIM-19, a component of complex I of the ETC, and may directly affect mitochondrial function and ROS generation. Induction of various myogenic transcription factors and muscle-specific E3 ubiquitin ligases (MURF-1 and atrogin-1) and activation of calpain, caspase 9, and caspase 3 can contribute to muscle atrophy and proteolytic cleavage of myofilament proteins. Mitochondrial dysfunction may also lead to ROS-mediated modification of myofilament proteins in a manner that negatively affects contractile function.

In summary, the findings presented in this article implicate JAK/STAT3 signaling in the development of diaphragm atrophy, mitochondrial dysfunction, and weakness during CMV (**Fig. 8**). Since the extended weaning period caused by VIDD contributes to the clinical risk and cost burden for a large number of ICU patients, inhibition of JAK/STAT3 signaling provides a promising therapeutic strategy for addressing this clinically important condition.

## Supplementary Material

Supplemental Data

## References

[1] Wunsch H. Linde-Zwirble W. T. Angus D. C. Hartman M. E. Milbrandt E. B. Kahn J. M. (2010) The epidemiology of mechanical ventilation use in the United States. Crit. Care Med. 38, 1947–1953 20639743 10.1097/CCM.0b013e3181ef4460

[2] Esteban A. Anzueto A. Alia I. Gordo F. Apezteguia C. Palizas F. Cide D. Goldwaser R. Soto L. Bugedo G. Rodrigo C. Pimentel J. Raimondi G. Tobin M. J. (2000) How is mechanical ventilation employed in the intensive care unit? An international utilization review. Am. J. Respir. Crit. Care Med. 161, 1450–1458 10806138 10.1164/ajrccm.161.5.9902018

[3] Esteban A. Anzueto A. Frutos F. Alia I. Brochard L. Stewart T. E. Benito S. Epstein S. K. Apezteguia C. Nightingale P. Arroliga A. C. Tobin M. J. (2002) Characteristics and outcomes in adult patients receiving mechanical ventilation: a 28-day international study. JAMA 287, 345–355 11790214 10.1001/jama.287.3.345

[4] Vassilakopoulos T. Petrof B. J. (2004) Ventilator-induced diaphragmatic dysfunction. Am. J. Respir. Crit. Care Med. 169, 336–341 14739134 10.1164/rccm.200304-489CP

[5] Levine S. Nguyen T. Taylor N. Friscia M. E. Budak M. T. Rothenberg P. Zhu J. Sachdeva R. Sonnad S. Kaiser L. R. Rubinstein N. A. Powers S. K. Shrager J. B. (2008) Rapid disuse atrophy of diaphragm fibers in mechanically ventilated humans. N. Engl. J. Med. 358, 1327–1335 18367735 10.1056/NEJMoa070447

[6] Jaber S. Petrof B. J. Jung B. Chanques G. Berthet J. P. Rabuel C. Bouyabrine H. Courouble P. Koechlin-Ramonatxo C. Sebbane M. Similowski T. Scheuermann V. Mebazaa A. Capdevila X. Mornet D. Mercier J. Lacampagne A. Philips A. Matecki S. (2011) Rapidly progressive diaphragmatic weakness and injury during mechanical ventilation in humans. Am. J. Respir. Crit. Care Med. 183, 364–371 20813887 10.1164/rccm.201004-0670OC

[7] De Jonghe B. Bastuji-Garin S. Durand M. C. Malissin I. Rodrigues P. Cerf C. Outin H. Sharshar T. (2007) Respiratory weakness is associated with limb weakness and delayed weaning. Crit. Care Med. 35, 2007–2015 17855814 10.1097/01.ccm.0000281450.01881.d8

[8] Hermans G. Agten A. Testelmans D. Decramer M. Gayan-Ramirez G. (2010) Increased duration of mechanical ventilation is associated with decreased diaphragmatic force: a prospective observational study. Crit. Care Med. 14, R127 10.1186/cc9094 PMC2945090 20594319

[9] Esteban A. Frutos F. Tobin M. J. Alia I. Solsona J. F. Inmaculada V. Fernandez R. De La Cal M. A. Benito S. Tomas R. Carriedo D. Macias S. Blanco J. (1995) A comparison of four methods of weaning patients from mechanical ventilation. N. Engl. J. Med. 332, 345–350 7823995 10.1056/NEJM199502093320601

[10] Powers S. K. Hudson M. B. Nelson W. B. Talbert E. E. Min K. Szeto H. H. Kavazis A. N. Smuder A. J. (2012) Mitochondria-targeted antioxidants protect against mechanical ventilation-induced diaphragm weakness. Crit. Care Med. 39, 1749–1759 21460706 10.1097/CCM.0b013e3182190b62 PMC4995067

[11] Ochala J. Renaud G. Diez M. L. Banduseela V. C. Aare S. Ahlbeck K. Radell P. J. Eriksson L. I. Larsson L. (2011) Diaphragm muscle weakness in an experimental porcine intensive care unit model. PLoS One 6, e20558 21698290 10.1371/journal.pone.0020558 PMC3115952

[12] Dasta J. F. McLaughlin T. P. Mody S. H. Piech C. T. (2005) Daily cost of an intensive care unit day: the contribution of mechanical ventilation. Crit. Care Med. 33, 1266–1271 15942342 10.1097/01.ccm.0000164543.14619.00

[13] Whidden M. A. Smuder A. J. Wu M. Hudson M. B. Nelson W. B. Powers S. K. (2010) Oxidative stress is required for mechanical ventilation-induced protease activation in the diaphragm. J. Appl. Physiol. 108, 1376–1382 20203072 10.1152/japplphysiol.00098.2010 PMC2867537

[14] McClung JM Kavazis A. N. Whidden M. A. DeRuisseau K. C. Falk D. J. Criswell D. S. Powers S. K. (2007) Antioxidant administration attenuates mechanical ventilation-induced rat diaphragm muscle atrophy independent of protein kinase B (PKB-Akt) signalling. J. Physiol. 585, 203–215 17916612 10.1113/jphysiol.2007.141119 PMC2375462

[15] Betters J. L. Criswell D. S. Shanely R. A. Van Gammeren D. Falk D. Deruisseau K. C. Deering M. Yimlamai T. Powers S. K. (2004) Trolox attenuates mechanical ventilation-induced diaphragmatic dysfunction and proteolysis. Am. J. Respir. Crit. Care Med. 170, 1179–1184 15374845 10.1164/rccm.200407-939OC

[16] McClung J. M. Kavazis A. N. Deruisseau K. C. Falk D. J. Deering M. A. Lee Y. Sugiura T. Powers S. K. (2007) Caspase-3 regulation of diaphragm myonuclear domain during mechanical ventilation-induced atrophy. Am. J. Respir. Crit. Care Med. 175, 1150–1159 10.1164/rccm.200601-142OC PMC1899279 17082496

[17] Smuder A. J. Kavazis A. N. Hudson M. B. Nelson W. B. Powers S. K. (2010) Oxidation enhances myofibrillar protein degradation via calpain and caspase-3. Free Radic. Biol. Med. 49, 1152–1160 20600829 10.1016/j.freeradbiomed.2010.06.025 PMC2930052

[18] Tang H. Lee M. Budak M. T. Pietras N. Hittinger S. Vu M. Khuong A. Hoang C. D. Hussain S. N. A. Levine S. Shrager J. B. (2011) Intrinsic apoptosis in mechanically ventilated human diaphragm: linkage to a novel Fos/FoxO1/STAT3-Bim axis. FASEB J. 25, 2921–2936 21597002 10.1096/fj.11-183798 PMC3157683

[19] Kavazis A. N. Talbert E. E. Smuder A. J. Hudson M. B. Nelson W. B. Powers S. K. (2009) Mechanical ventilation induces diaphragmatic mitochondrial dysfunction and increased oxidant production. Free Radic. Biol. Med. 46, 842–850 19185055 10.1016/j.freeradbiomed.2009.01.002 PMC2906125

[20] White J. P. Baltgalvis K. A. Puppa M. J. Sato S. Baynes J. W. Carson J. A. (2011) Muscle oxidative capacity during IL-6-dependent cancer cachexia. Am. J. Physiol. Regul. Integr. Comp. Physiol. 300, R201–R211 21148472 10.1152/ajpregu.00300.2010 PMC3043802

[21] Deuse T. Hua X. Taylor V. Stubbendorff M. Baluom M. Chen Y. Park G. Velden J. Streichert T. Reichenspurner H. Robbins R. C. Schrepfer S. (2012) Significant reduction of acute cardiac allograft rejection by selective Janus kinase-1/3 inhibition using R507 and R545. Transplantation 94, 695–702 22971540 10.1097/TP.0b013e3182660496

[22] Meyer D. M. Jesson M. I. Li X. Elrick M. M. Funckes-Shippy C. L. Warner J. D. Gross C. J. Dowty M. E. Ramaiah S. K. Hirsch J. L. Saabye M. J. Barks J. L. Kishore N. Morris D. L. (2010) Anti-inflammatory activity and neutrophil reductions mediated by the JAK1/JAK3 inhibitor, CP-690,550, in rat adjuvant-induced arthritis. J. Inflamm. (Lond.) 7, 41 20701804 10.1186/1476-9255-7-41 PMC2928212

[23] Moresi V. Williams A. H. Meadows E. Flynn J. M. Potthoff M. J. McAnally J. Shelton J. M. Backs J. Klein W. H. Richardson J. A. Bassel-Duby R. Olson E. N. (2010) Myogenin and Class II HDACs control neurogenic muscle atrophy by inducing E3 ubiquitin ligases. Cell 143, 35–45 20887891 10.1016/j.cell.2010.09.004 PMC2982779

[24] Maes K. Testelmanns D. Powers S. Decramer M. Gayan-Ramirez G. (2007) Leupeptin inhibits ventilator-induced diaphragm dysfunction in rats. Am. J. Respir. Crit. Care Med. 175, 1134–1138 17379854 10.1164/rccm.200609-1342OC

[25] Wegrzyn J. Potla R. Chwae Y. J. Sepuri N. B. Zhang Q. Koeck T. Derecka M. Szczepanek K. Szelag M. Gornicka A. Moh A. Moghaddas S. Chen Q. Bobbili S. Cichy J. Dulak J. Baker D. P. Wolfman A. Stuehr D. Hassan M. O. Fu X. Y. Avadhani N. Drake J. I. Fawcett P. Lesnefsky E. J. Larner A. C. (2009) Function of mitochondrial Stat3 in cellular respiration. Science 323, 793–797 19131594 10.1126/science.1164551 PMC2758306

[26] Tammineni P. Anugula C. Mohammed F. Anjaneyulu M. Larner A. Sepuri N. B. (2013) The import of the transcription factor STAT3 into mitochondria depends on GRIM-19, a component of the electron transport chain. J. Biol. Chem. 288, 4723–4732 23271731 10.1074/jbc.M112.378984 PMC3576077

[27] Gough D. J. Corlett A. Schlessinger K. Wegrzyn J. Larner A. C. Levy D. E. (2009) Mitochondrial STAT3 supports Ras-dependent oncogenic transformation. Science 324, 1713–1716 19556508 10.1126/science.1171721 PMC2840701

[28] Shulga N. Pastorino J. G. (2012) GRIM-19 mediated translocation of STAT3 to mitochondria is necessary for TNF induced necroptosis. J. Cell Sci. 125, 2995–3003 22393233 10.1242/jcs.103093 PMC3434811

[29] Zhou L. Too H. P. (2011) Mitochondrial localized STAT3 is involved in NGF induced neurite outgrowth. PLoS One 6, e21680 21738764 10.1371/journal.pone.0021680 PMC3124549

[30] Song B. Jin H. Yu X. Zhang Z. Yu H. Ye J. Xu Y. Zhou T. Oudit G. Y. Ye J. Y. Chen C. Gao P. Zhu D. Penninger J. M. Zhong J. C. (2013) Angiotensin-converting enzyme 2 attenuates oxidative stress and VSMC proliferation via the JAK2/STAT3/SOCS3 and profilin-1/MAPK signaling pathways. Regul. Pept. 185, 44–51 23816468 10.1016/j.regpep.2013.06.007

[31] Zhang Q. Raje V. Yakovlev V. A. Yacoub A. Szczepanek K. Meier J. Derecka M. Chen Q. Hu Y. Sisler J. Hamed H. Lesnefsky E. J Jr. Valeriem K. Dent P. Larner A. C. (2013) Mitochondrial-localized Stat3 promotes breast cancer growth via phosphorylation of Serine 727. J. Biol. Chem. 288, 31280–31288 24019511 10.1074/jbc.M113.505057 PMC3829438

[32] Szczepanek K. Chen Q. Derecka M. Salloum F. N. Zhang Q. Szelag M. Cichy J. Kukreja R. C. Dulak J. Lesnefsky E. J. Larner A. C. J. (2011) Mitochondrial-targeted Signal transducer and activator of transcription 3 (STAT3) protects against ischemia-induced changes in the electron transport chain and the generation of reactive oxygen species. J. Biol. Chem. 286, 29610–29620 21715323 10.1074/jbc.M111.226209 PMC3191002

[33] Reid M. B. Moylan J. S. (2011) Beyond atrophy: redox mechanisms of muscle dysfunction in chronic inflammatory disease. J. Physiol. 589, 2171–2179 21320886 10.1113/jphysiol.2010.203356 PMC3098696

[34] Lokireddy S. McFarlane C. Ge X. Zhang H. Sze S. K. Sharma M. Kambadur R. (2011) Myostatin induces degradation of sarcomeric proteins through a Smad3 signaling mechanism during skeletal muscle wasting. Mol. Endocrinol. 25, 1936–1949 21964591 10.1210/me.2011-1124 PMC5417176

[35] Polge C. Heng A. E. Jarzaguet M. Ventadour S. Claustre A. Combaret L. Béchet D. Matondo M. Uttenweiler-Joseph S. Monsarrat B. Attaix D. Taillandier D. (2011) Muscle actin is polyubiquitinylated in vitro and in vivo and targeted for breakdown by the E3 ligase MuRF1. FASEB J. 25, 3790–3802 21764995 10.1096/fj.11-180968

[36] Bodine S. C. Latres E. Baumhueter S. Lai V. K. Nunez L. Clarke B. A. Poueymirou W. T. Panaro F. J. Na E. Dharmarajan K. Pan Z. Q. Valenzuela D. M. DeChiara T. M. Stitt T. N. Yancopoulos G. D. Glass D. J. (2001) Identification of ubiquitin ligases required for skeletal muscle atrophy. Science 294, 1704–1708 11679633 10.1126/science.1065874

[37] Kim J. Won K. J. Lee H. M. Hwang B. Y. Bae Y. M. Choi W. S. Song H. Lim K. W. Lee C. K. Kim B. (2009) p38 MAPK participates in muscle-specific RING finger 1-mediated atrophy in cast-immobilized rat gastrocnemius muscle. Korean J. Physiol. Pharmacol. 13, 491–496 20054497 10.4196/kjpp.2009.13.6.491 PMC2802311

[38] Moresi V. Williams A. H. Meadows E. Flynn J. M. Potthoff M. J. McAnally J. Shelton J. M. Backs J. Klein W. H. Richardson J. A. Bassel-Duby R. Olson E. N. (2010) Myogenin and class II HDACs control neurogenic muscle atrophy by inducing E3 ubiquitin ligases. Cell 143, 35–45 20887891 10.1016/j.cell.2010.09.004 PMC2982779

[39] Yokogami K. Wakisaka S. Avruch J. Reeves S. A. (2000) Serine phosphorylation and maximal activation of STAT3 during CNTF signaling is mediated by the rapamycin target mTOR. Curr. Biol. 10, 47–50 10660304 10.1016/s0960-9822(99)00268-7

[40] Jain N. Zhang T. Kee W. H. Li W. Cao X. (1999) Protein kinase C delta associates with and phosphorylates Stat3 in an interleukin-6-dependent manner. J. Biol. Chem. 274, 24392–24400 10446219 10.1074/jbc.274.34.24392

[41] Chung J. Uchida E. Grammer T. C. Blenis J. (1997) STAT3 serine phosphorylation by ERK-dependent and -independent pathways negatively modulates its tyrosine phosphorylation. Mol. Cell. Biol. 17, 6508–6516 9343414 10.1128/mcb.17.11.6508 PMC232504

[42] Turkson J. Bowman T. Adnane J. Zhang Y. Djeu J. Y. Sekharam M. Frank D. A. Holzman L. B. Wu J. Sebti S. Jove R. (1999) Requirement for Ras/Rac1-mediated p38 and c-Jun N-terminal kinase signaling in Stat3 transcriptional activity induced by the Src oncoprotein. Mol. Cell. Biol. 19, 7519–7528 10523640 10.1128/mcb.19.11.7519 PMC84756

[43] Kurdi M. Booz G. W. (2009) JAK redux: a second look at the regulation and role of JAKs in the heart. Am. J. Physiol. Heart Circ. Physiol. 297, H1545–H1556 19717737 10.1152/ajpheart.00032.2009 PMC2781365

