# Supplementary material for: Inhibition of Janus kinase signaling during controlled mechanical ventilation prevents ventilation-induced diaphragm dysfunction
Source: FASEB J. 2014 Jul;28(7):2790–803. doi: 10.1096/fj.13-244210 (PMC4062832; doi:10.1096/fj.13-244210)
Supplement: Supplemental Data [file supp_28_7_2790__index.html]

Inhibition of Janus kinase signaling during controlled mechanical ventilation prevents ventilation-induced diaphragm dysfunction — Inhibition of Janus kinase signaling during controlled mechanical ventilation prevents ventilation-induced diaphragm dysfunction — Supplemental Data 

# Inhibition of Janus kinase signaling during controlled mechanical ventilation prevents ventilation-induced diaphragm dysfunction

## Supplemental Data

**Files in this Data Supplement:**

- Supplemental Data - (*13-244210SuppData.zip; compressed file 221 KB*)
