# Supplementary material for: Inhibition of Janus kinase signaling during controlled mechanical ventilation prevents ventilation-induced diaphragm dysfunction
Source: FASEB J. 2014 Jul;28(7):2790–803. doi: 10.1096/fj.13-244210 (PMC4062832; doi:10.1096/fj.13-244210)
Supplement: Supplemental Data [file supp_fj.13-244210_13-244210SuppData.zip › VIDD-Supplemental-Figure1.pdf]

Supplemental Figure 1

Signaling Kinase(s)	Cell-based assay	R507 EC50 (nM)	Ratio
JAK1, JAK3	Primary T-cell: IL-2 proliferation	0.021	23
JAK2	CHEPs: Erythropoietin Survival	0.49	
JAK1, JAK3	Whole Blood lymphocytes: IL-2 induced pSTAT5	0.417	78
JAK2	Whole Blood granulocytes: GM-CSF induced pSTAT5	32.7	

Supplemental Figure 1

R548 is a JAK1/JAK3 inhibitor with substantial selectivity over JAK2-mediated signaling in cell-based assays. Cell based assays were utilized to assess compound potency and selectivity. R548 is a prodrug that is bioconverted to R507 in vivo and R507 was therefore utilized in cell based assays for EC50 determinations. Interleukin-2 (IL-2) drives primary human T-cell proliferation through JAK1 and JAK3. Erythropoietin (EPO) signaling through JAK2 is required for human primary Cultured Erythroid Progenitor Cell (CHEP) differentiation and survival.. IL-2 stimulates phosphorylation of STAT5 by JAK1 and JAK3 in blood lymphocytes. Granulocyte-macrophage colony-stimulating factor (GM-CSF) stimulates JAK2-dependent STAT5 phosphorylation in blood granulocytes. Table adapted from Deuse et al., 2012 (ref. 21).
