# Supplementary material for: Inhibition of Janus kinase signaling during controlled mechanical ventilation prevents ventilation-induced diaphragm dysfunction
Source: FASEB J. 2014 Jul;28(7):2790–803. doi: 10.1096/fj.13-244210 (PMC4062832; doi:10.1096/fj.13-244210)
Supplement: Supplemental Data [file supp_fj.13-244210_13-244210SuppData.zip › VIDD-Supplemental-Figure2.pdf]

Supplemental Figure 2

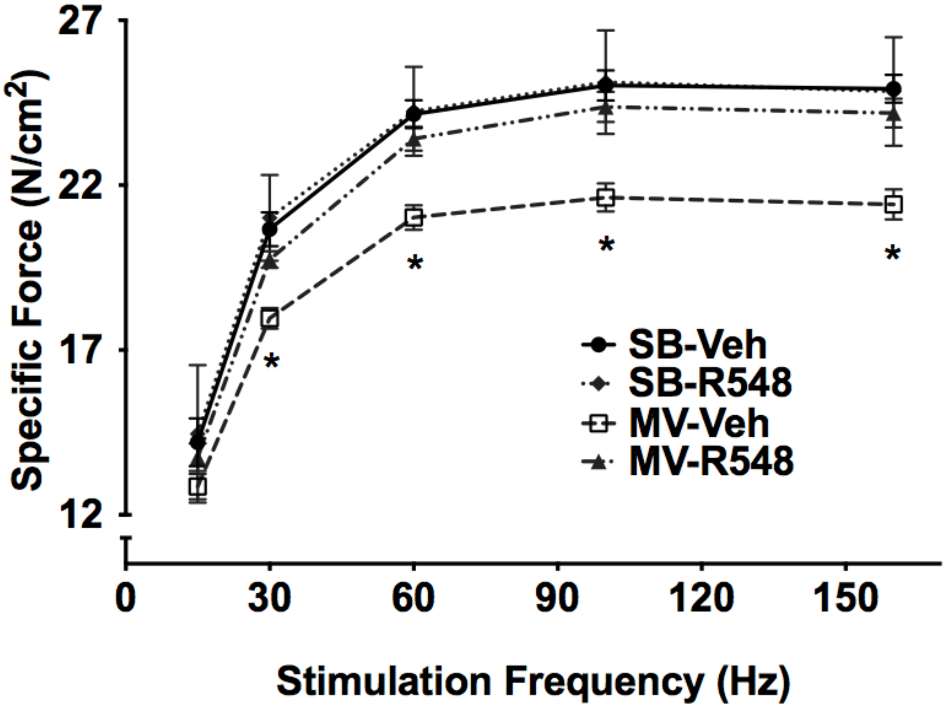

Supplemental Figure 2

Treatment with the JAK1/JAK3 inhibitor R548 (3 mg/kg/hr) prevents controlled mechanical ventilation-induced contractile dysfunction in the diaphragm in a 12-hour model. Diaphragm strip *ex-vivo f*orce-frequency relationship. Results are means ± SEM with n = 10 per group. *P* values calculated versus vehicle control group using one-way ANOVA (Tukey's post-hoc analysis). * $P < 0.05$ MV-Veh vs all other groups.
